# A microfluidic device for isolating intact chromosomes from single mammalian cells and probing their folding stability by controlling solution conditions

**DOI:** 10.1038/s41598-018-31975-5

**Published:** 2018-09-12

**Authors:** Tomohiro Takahashi, Kennedy O. Okeyo, Jun Ueda, Kazuo Yamagata, Masao Washizu, Hidehiro Oana

**Affiliations:** 1 Department of Mechanical Engineering, The University of Tokyo, Tokyo, 113-8656 Japan; 2 Institute for Frontier Life & Medical Sciences, Kyoto University, Kyoto, 606-8507 Japan; 3 Centre for Advanced Research and Education, Asahikawa Medical University, Asahikawa, 078-8510 Japan; 4 Faculty of Biology-Oriented Science and Technology, KINDAI University, Kinokawa, 649-6493 Japan; 5 Department of Bioengineering, The University of Tokyo, Tokyo, 113-8656 Japan

## Abstract

Chromatin folding shows spatio-temporal fluctuations in living undifferentiated cells, but fixed spatial heterogeneity in differentiated cells. However, little is known about variation in folding stability along the chromatin fibres during differentiation. In addition, effective methods to investigate folding stability at the single cell level are lacking. In the present study, we developed a microfluidic device that enables non-destructive isolation of chromosomes from single mammalian cells as well as real-time microscopic monitoring of the partial unfolding and stretching of individual chromosomes with increasing salt concentrations under a gentle flow. Using this device, we compared the folding stability of chromosomes between non-differentiated and differentiated cells and found that the salt concentration which induces the chromosome unfolding was lower (≤500 mM NaCl) for chromosomes derived from undifferentiated cells, suggesting that the chromatin folding stability of these cells is lower than that of differentiated cells. In addition, individual unfolded chromosomes, i.e., chromatin fibres, were stretched to 150–800 µm non-destructively under 750 mM NaCl and showed distributions of highly/less folded regions along the fibres. Thus, our technique can provide insights into the aspects of chromatin folding that influence the epigenetic control of cell differentiation.

## Introduction

In eukaryotic cells, genomic DNA bound to histones is folded and stored in the nucleus. Cellular activity is maintained by the expression of genes at the appropriate place and time, which requires the partial loosening of DNA–histone complexes. Since the control of gene expression involves chemical modifications of DNA bases and histones that alter the folding stability (loosening or tightening) of the chromatin at specific sites, gene expression profiles vary according to cell type and differentiation status^1^. Transcriptional activity differs among allogeneic cells^2–4^, and cancerous tissues harbour a mixed population of cells with distinct expression profiles^5^. As such, there is a need for a technique that enables epigenetic analyses at the single-cell level to evaluate the relationship between the distribution of chemical modifications of DNA or histones and the folding stability of chromatin as well as gene expression profiles. This information can provide insight into the mechanisms by which a state of differentiation is induced or maintained and how these mechanisms contribute to cancer development.

Micrococcal nuclease sequencing, chromatin conformation capture sequencing, assay for transposase-accessible chromatin by high-throughput sequencing, and chromatin immunoprecipitation sequencing are analytical methods that can be used to identify DNA sites that lack or harbour loosely bound histones or that are bound by specific proteins at a single-base resolution^6–10^. However, since these approaches involve a DNA fragmentation step prior to sequencing and utilise short read sequences, it is difficult to obtain information about higher-order DNA structure and folding stability. In addition, whole-genome coverage is low when these methods are applied to single cells due to sample loss during preparation^11^.

Immunofluorescence labelling of chromosomes is another epigenetic analysis technique^12^ that can be applied to single cells. In this method, chromosomes are spread out on a glass substrate near the source cells, which are seeded on the substrate with adequate spacing. However, this approach does not provide high-resolution information about the distribution of chemical modifications or folding stability along chromatin fibres. In addition, it is difficult to investigate changes in the higher-order folding structure resulting from alterations in the conditions of the surrounding solution—which alter the strength of interactions between DNA and DNA-binding proteins—due to the adsorption of chromosomes onto the glass substrate. Consequently, a technique that allows for the examination of chromosomes isolated from single cells without fragmentation and adsorption onto a substrate is needed.

Studies pioneering the use of single cell- and single chromosome-based techniques to investigate the properties of chromosomes have involved the extraction of mitotic chromosomes from mammalian/amphibian cells in an open cell culture dish under a microscope using micromanipulator-assisted micro-needles/-pipettes^13,14^. This approach has revealed the reversible condensation/decondensation of mitotic chromosomes by exposure to various cationic solutions in the open dish. However, this method has not been used to determine the correlation between the differentiation state of cells and the distribution of chromosome/chromatin folding stability. This lack of investigation may be attributed to practical challenges, e.g., sequential solution exchanges and the precise control of solution conditions in the open dish during the micromanipulation of cells/chromosomes.

Recently, microfluidic devices have been utilised in single cell/molecule-level biochemical analyses/experiments^15–18^. A characteristic feature of microfluidic devices is their ability to precisely control solution conditions by introducing the solution of interest into microfluidic channels. Although such devices have been used for various types of bioanalysis, methods for investigating chromatin/chromosomes, i.e., the complex of DNA and proteins, in single cells are less developed than those used for single-cell genome-wide gene expression analyses in which the analyte is basically naked fragmented DNA. To date, nano-/microfluidic channel devices for chromosome/large genomic DNA analysis that have been developed employ off-chip-prepared chromosomes/genomic DNA and have not yet been used for single cell-based experiments^19–21^.

We recently developed a method for isolating intact chromatin fibres from individual fission yeast cells that were then tethered to a microstructure for optical mapping after immunofluorescence labelling^22^ or for the evaluation of changes in chromatin folding in a solution that alters the strength of interactions between chromatin and associated proteins using microfluidic channels^23^. In this study, we further developed this microfluidic system and used it to compare the stability of chromatin folding in mammalian cells at various stages of differentiation (undifferentiated vs. differentiated). We found that the differentiation state was correlated with the folding stability of chromosomes. This is the first report of a method for isolating a chromosome in its native form from a target mammalian cell for single-molecule biochemical experiments using a microfluidic channel under a microscope. This technique can provide information concerning the distribution of chromatin folding stability along individual chromatin fibres. It will therefore be useful for single-cell/whole-genome analyses of chromatin, which are expected to provide novel insights into the epigenetic control of gene expression.

## Results

### A microfluidic device with an optically-driven microtool for the extraction of chromosomes and investigation of their stability

**Figure 1:**
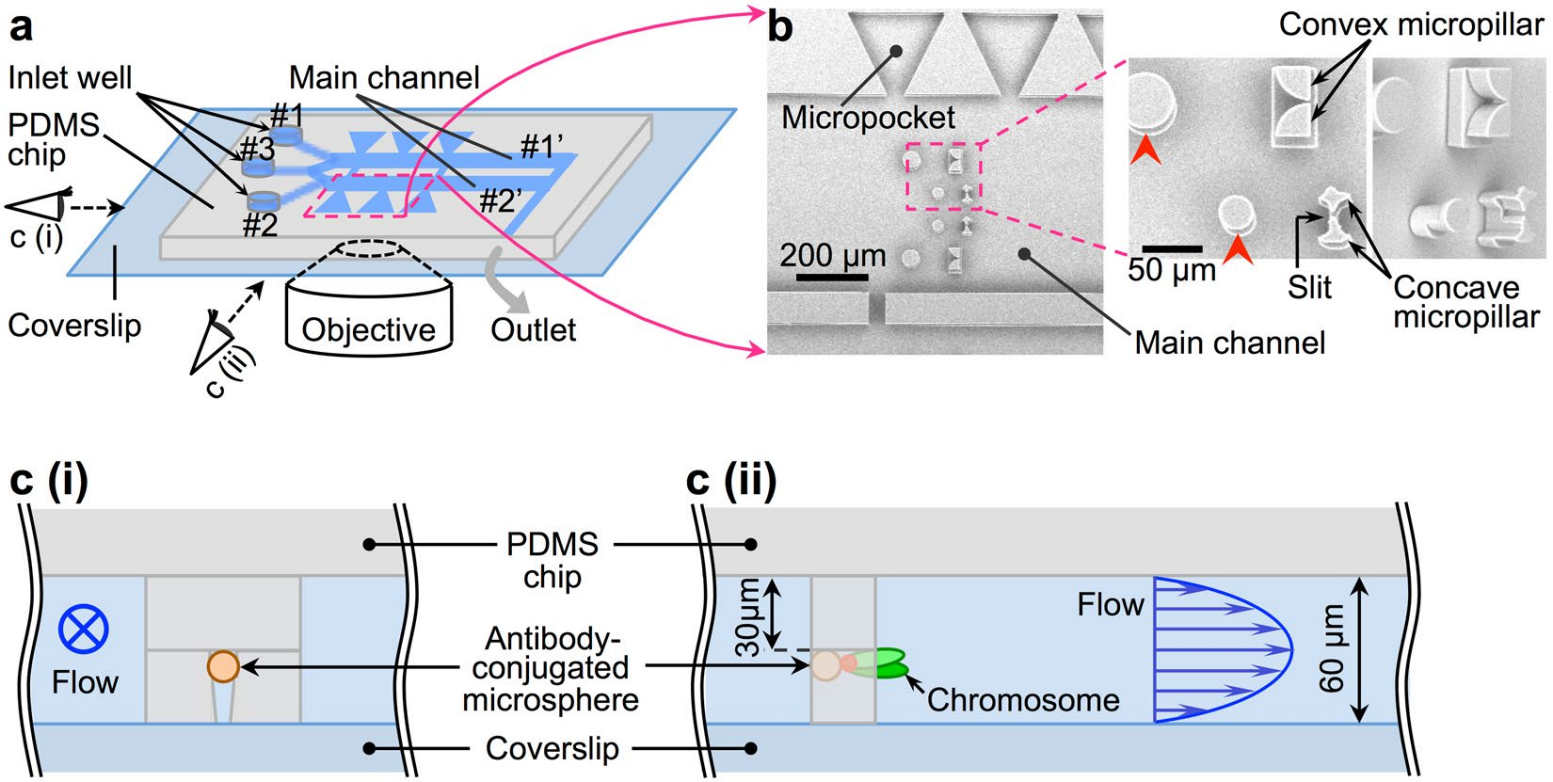
Microfluidic polydimethylsiloxane (PDMS) device for chromosome extraction and tethering. (**a**) Schematic illustration of the microfluidic device. The microfabricated PDMS chip (~2 mm thick) with micrometre-scale grooves on one side surface was bonded to a coverslip to form enclosed microchannels. At one end of the microchannels, through-holes (inner diameter: 2.5 mm) were made by punching the PDMS chip to obtain inlet wells. The other end of the microchannels was connected to a silicone tube to form an outlet. Solutions were dropped into the inlet wells and drawn into the main channels by creating suction through the silicone tube. The size of the main channel was as follows: width = 600 μm, height = 60 μm, length = ca. 6 mm. The schematic is not drawn to scale. (**b**) Scanning electron micrograph of the main channel region of the PDMS chip. Enlarged pictures: a pair of concave micropillars was used in this study. A pair of convex micropillars, first employed in our previous report^22^, was fabricated as a supplementary additional microstructure in the vicinity of thinner concave micropillars and was used as a reference in examining exposure and baking conditions for the fabrication of the mould. Circular micropillars (indicated by red arrowheads) were set at the upper stream side of the slit between another pair of micropillars (used for chromosome tethering) to prevent clogging of the slit by the introduced cells and antibody-conjugated microspheres. (**c** (i)) Schematic side view of the main channel around the micropillars in a fork-shaped structure for capturing the antibody-conjugated microsphere. The flow direction is from the front of the page to the back, and an antibody-conjugated microsphere is caught in the slit between the micropillars. The gap between micropillars was set to 3 or 5 μm, which was smaller than the diameter of the antibody-conjugated microspheres. (**c** (ii)) Schematic side view of the main channel around the micropillars with the trapped antibody-conjugated microsphere and chromosome. The schematics are not drawn to scale.

For the isolation and investigation of chromosomes from single mammalian cells, a microfluidic device was assembled by bonding a microfabricated polydimethylsiloxane (PDMS, commonly known as silicone rubber) chip onto a coverslip (Fig. 1a). The PDMS chip was fabricated using standard soft lithography^24^, as described in the Supplementary Material. The device had two main channels with micropockets along the side walls and micropillars on the floor of the channels (Fig. 1b). The micropockets served as reaction chambers in which solution conditions were altered by introducing different solutions into the main channels, with solutes diffusing between the main channels and the micropockets^25–28^. The basic structure of the device was similar to the one reported in our previous study, which focused on yeast spheroplasts^22^. However, since mammalian cells are much larger (diameter: ca. 20–30 µm), the height of the microchannel was increased to 60 µm and larger micropockets were created (entrance width: 50 µm, depth: 250 µm, length of the trapezoid base: 290 µm). Micropillars were used to tether isolated chromosomes by suspending chromosome-attached antibody-conjugated microspheres within the slit between the micropillars against the solution flow in the main channels^22^, which enabled direct observation of changes in the higher-order structures of the isolated chromosomes. It should be noted that the micropillars appear fork-shaped when viewed from the side, but concave or convex (fan-shaped) from viewed from underneath, through the coverslip of the device (Fig. 1b right, 1c (i)). Here, the gap between the micropillars was slightly larger from the coverslip (floor) side to PDMS (ceiling) side (Fig. 1c(i)), which was due to our photolithographic exposure system. Consequently, microspheres were usually captured at the base of micropillars under the flow (Fig. 1c(i), c(ii)). This ensures that the tethered chromosomes are in a suspended position away from the PDMS ceiling to prevent non-specific binding while permitting maximum exposure to the flow (assuming a plane Poiseuille flow with maximum flow velocity at the half height of the microchannel). Here, emphasis was on achieving chromosome suspension away from the channel ceiling/floor; the shape of the micropillars was of little importance. To realise this, in the present study, we employed fork-shaped micropillars to achieve both tethering and suspension of the chromosomes within the microchannel, allowing for the direct observation of changes in their higher-order structures. In our previous work, we used convex micropillars with slits for trapping antibody-conjugated microspheres attached to individual chromosomes^22^. Since the fabrication of the convex micropillars was already established in our previous work, we used them as references for assessing the successful fabrication of the concave micropillars used in the present study. For this reason, in Fig. 1b, the two types of pairs of micropillars appear adjacent to one another within the new microfluidic device. It should be noted that, in comparison to the convex micropillars, the newly-designed concave micropillars used in this study improved the visibility of the chromosome, especially around the tethering point. The detailed size and layout of the microstructures are described in the Supplementary Material (Figure S1).

**Figure 2:**
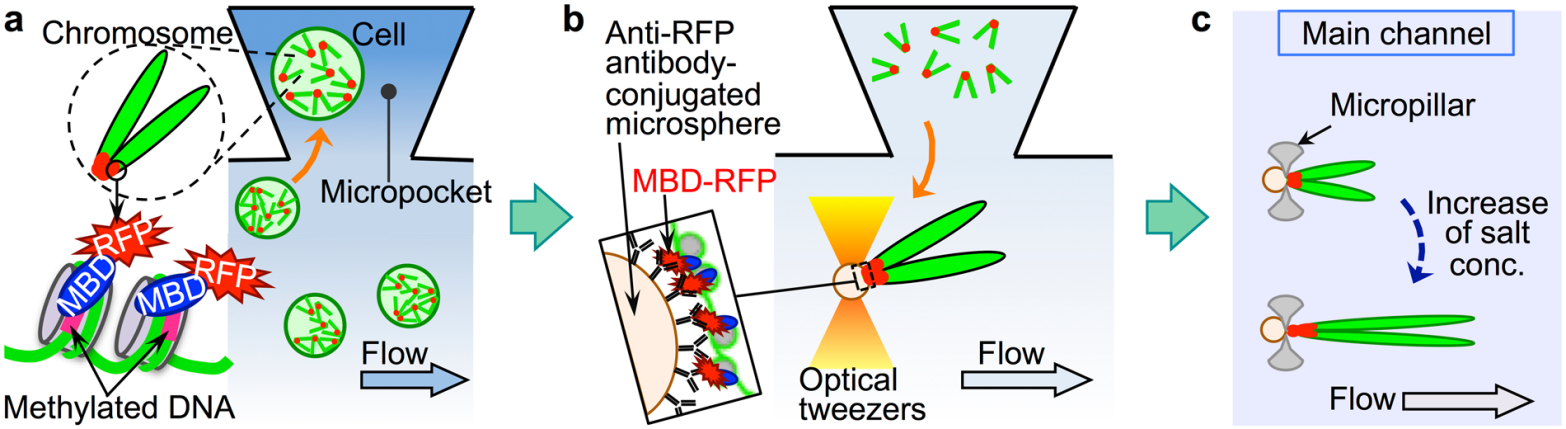
Schematic illustrations (top view) of the principle underlying single cell- and single chromosome-level experiments. (**a**) Translocation of a cell in M phase from the main channel into the micropocket using optical tweezers. In cells from Methyl*RO* mice, methylated regions of DNA were identified by the presence of methyl-CpG-binding domain-red fluorescent protein (MBD-RFP) fusion proteins. (**b**) Individual chromosomes were extracted from single cells by subjecting the cells to osmotic shock, and some of the released chromosomes were captured and translocated individually to the micropillar region for tethering using optically-driven antibody-conjugated microspheres. (**c**) Translocated chromosomes were tethered to the slit between micropillars via antibody-conjugated microspheres whose diameter is slightly larger than the gap of the slit. Solutions of interest were sequentially introduced into the main channels (e.g., solutions with stepwise increases in the salt concentration), and the responses, including changes in chromosome unfolding, were evaluated. The schematics are not drawn to scale.

Methyl*RO* mouse cells expressing a methyl-CpG-binding domain (MBD)-red fluorescent protein (RFP) fusion protein that binds to methylated DNA were used in these experiments (Fig. 2a)^29^. Cell suspension was placed into one of the three inlet wells (Fig. 1a, left end) and sucked into the main channels. A hypotonic solution or a solution of interest was then introduced through the two remaining ports. Using two ports helped to prevent backflow during solution exchange, and the selective use of the three ports reduced the influx of cell debris into the two main channels (details are described in the Methods section). To manipulate cells and chromosomes in the enclosed space of the microchannels and micropockets, optical tweezers were used. Optical tweezers use laser light to trap a micrometre-sized object at the laser focus point formed by an objective lens when the refractive index of the object is greater than that of the surrounding medium^30^. Using this method, cells and compacted chromosomes suspended in aqueous solution can be trapped and translocated individually in a non-destructive manner. However, unfolded chromosomes cannot be trapped by optical tweezers owing to their lower chromatin packing density. Hence, we developed an optically-driven microtool consisting of antibody-conjugated microspheres which can attach to a target protein associated with the chromatin, and which can be manipulated individually by optical tweezers^22^. By utilising the property of antigen–antibody binding, the micromanipulation of individual chromosomes through the microspheres was achieved. In this study, anti-RFP antibody-conjugated microspheres were prepared and used as the optically-driven microtool (Fig. 2b). It should be noted that the peri-centromeric region of mouse chromosomes contains hypermethylated DNA region^31,32^, and the short arms of mouse chromosomes are very short, i.e., chromosomes are telocentric, giving them a narrow V-shaped morphology. Thus, the peri-centromeric region where MBD-RFP proteins accumulate, i.e., the vertex of the V-shaped morphology, is easily captured using the antibody-conjugated microspheres.

The basic procedure for the manipulation of single cells and single chromosomes by the optically-driven microtool within the specially designed microfluidic device is as follows:

(I) Cells synchronised in M phase were introduced into the main channels of the microfluidic device and some cells were individually placed in separate micropockets using optical tweezers (Fig. 2a). Chromosomes—the highly compacted form of individual genomic DNA molecules—in M phase can be individually distinguished and manipulated.
(II) The main channels were flushed with isotonic solution to remove excess cells, after which a hypotonic solution was then introduced, causing cells to burst from osmotic shock; as a result, chromosomes were released from the cells. After chromosome extraction in each micropocket, antibody-conjugated microspheres were introduced into the main channels, and individual chromosomes were captured at the peri-centromeric region using optical tweezers. Captured chromosomes were transported from the micropockets to the main channel (Fig. 2b).
(III) Translocated chromosomes were then tethered to the slit between micropillars via the antibody-conjugated microspheres and exposed to solutions of interest, e.g., high-salt solutions, which were introduced into the main channels. Tethered chromosomes were oriented along the direction of the flow; therefore, when the chromosomes unfolded, the spatial resolution along the chromosomes was improved (Fig. 2c).

It should be noted that this tethering method utilising micropillars is suitable for observation of individual non-fragmented chromosomes/chromatins that span multiple frames of a video microscope.

### Extraction, translocation, and tethering of chromosomes from single MEFs and ES cells

**Figure 3:**
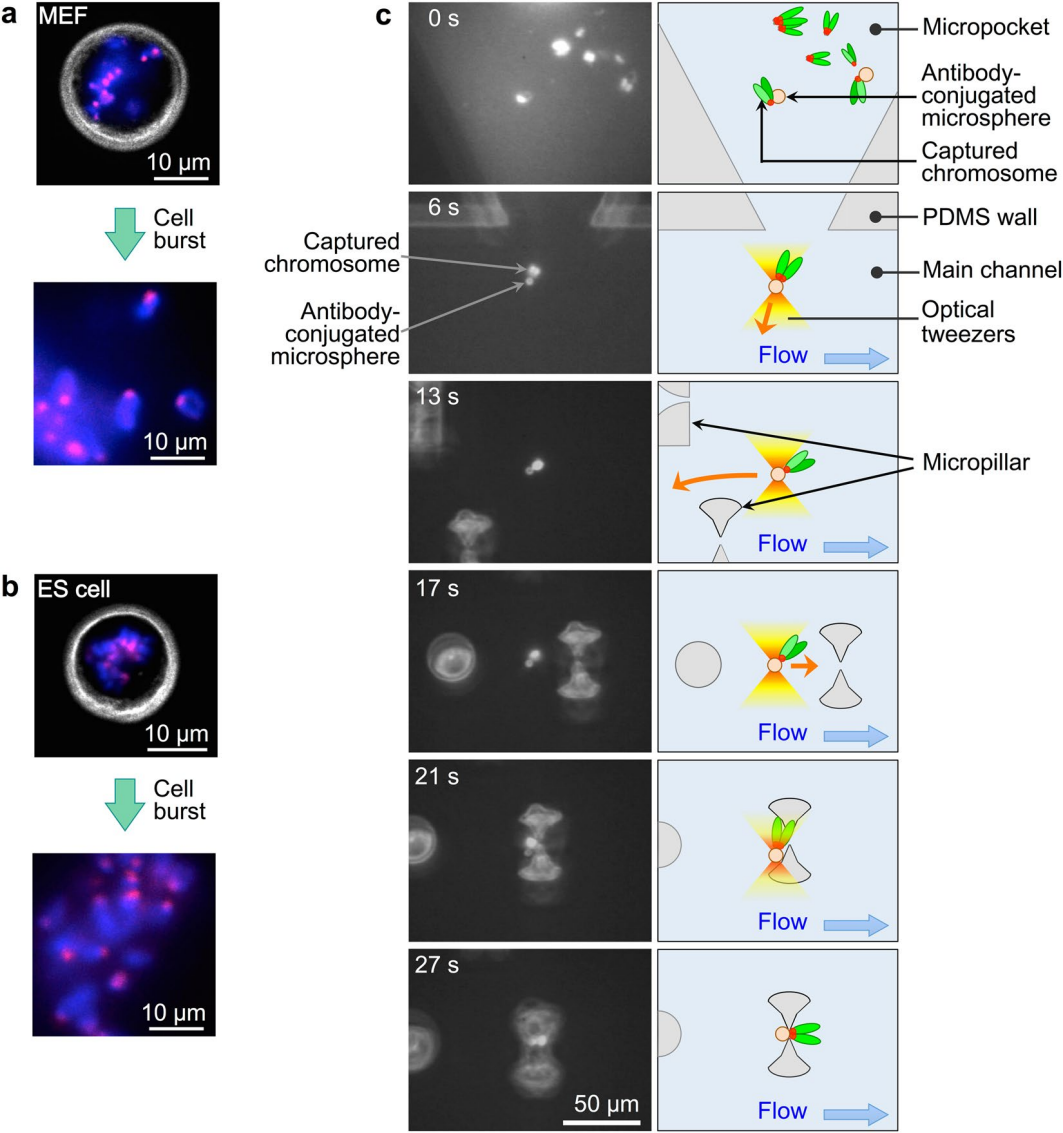
Single-cell chromosome extraction and chromosome tethering. (**a**,**b**) Pseudocoloured images of M phase cells in each micropocket and chromosomes isolated from each cell (bottom). DNA was stained with Hoechst 33342 (blue) and methyl-CpG-binding domain-red fluorescent protein (MBD-RFP) is visible as a red fluorescent signal, with grey representing a phase-contrast image of the cell membrane. Mouse embryonic fibroblasts (MEFs) (**a**) and embryonic stem (ES) cells (**b**) in isotonic solution (top) and chromosomes released from the burst cells in Triton X solution without salt (bottom). Images of isolated chromosomes were captured ca. 3 min after the cells burst. (**c**) Left: Representative fluorescence images showing the translocation and tethering of the MEF chromosome. The chromosomes were visualised by YO-PRO-1 and antibody-conjugated microspheres were visualised by auto-fluorescence. During the manipulation, bright-field illumination was simultaneously applied to visualise microstructures made from PDMS. The time at which the translocation of the chromosome was started was set to 0 s. Right: schematics corresponding to the images on the left. Orange arrows indicate the direction of translocation using optical tweezers. One of the isolated chromosomes was captured by the anti-RFP antibody-conjugated microsphere in the micropocket (0 s). Subsequently, the chromosome (a pair of chromatids) was dragged out of the micropocket using the antibody-conjugated microsphere driven by the optical tweezers (6 s) and transported to the micropillar region in the main channel (13 s). Then, the transported chromosome was moved to approach a slit between the micropillars upstream of the main channel (17 s). With the flow in the main channel, the free ends of the long arms of the trapped chromosome were forced through the slit (21 s). When passing through the slit, the optical trapping ceased, the microsphere moved between the pair of micropillars under flow, and tethering was achieved (27 s). A corresponding movie file is available in the Supplementary Material (Movie 2).

Chromosomes were extracted from mouse embryonic fibroblasts (MEFs) (Fig. 3a) and embryonic stem (ES) cells (Fig. 3b) in each micropocket by introducing a hypotonic solution into the main channels. This resulted in the swelling of cells, which eventually burst within 3–5 min, rapidly releasing intact chromosomes (Supplementary Movie 1). Soon after bursting, Triton X solution was introduced into the device for 10–15 min to lyse the cell membrane. As a result, some chromosomes became dispersed, while others remained attached to one other. In subsequent experiments, we focused on the separated chromosomes, which typically accounted for ≤10% of the total number of chromosomes. It should be noted that there was no major difference between MEFs and ES cells in terms of the success rate for the extraction of individual chromosomes. After the establishment of one-to-one binding between separated chromosomes and anti-RFP antibody-conjugated microspheres, Triton X solution containing anti-RFP antibody (2 µg/ml) was introduced into the main channels for 10–15 min to flush out excess microspheres, and to prevent multiple microspheres from binding to the captured chromosomes as well as the dissociation of MBD-RFP from methylated DNA as the salt concentration increased. Following this, the main channels were once again flushed with Triton X solution and chromosome tethering was performed.

Figure 3c shows an example of the translocation and tethering of a chromosome from a MEF (hereafter, MEF chromosomes). The corresponding movie file is available in the Supplementary Material (Movie 2). This tethering process took 5–10 min and 1–3 chromosomes were usually tethered individually at the micropillars. During translocation, the morphology of the captured MEF chromosomes remained unaltered, despite the shear force, and they maintained the typical telocentric chromosome shape (e.g., Fig. 3c left, 6 s).

Chromosomes extracted from ES cells (hereafter, ES cell chromosomes) swelled slightly during lysis of cell membrane with Triton X solution, becoming less compact. In fact, the captured chromosomes became unfolded and stretched (by 5–6 times, ~40 µm in contour length) during translocation using antibody-conjugated microspheres due to hydrodynamic shear force (Supplementary Movie 3), making it almost impossible to tether them to the slit between the micropillars from their trailing end. To stabilise ES cell chromosome morphology according to a chromosome sorting method^33^, we exposed extracted ES cell chromosomes to a Triton X solution containing 100 mM KCl. The addition of salt prevented the unfolding of chromosomes obtained from ES cells (Supplementary Movie 4) and facilitated their translocation and tethering, which was similar to that of MEF chromosomes.

### Folding stability of MEF chromosomes

**Figure 4:**
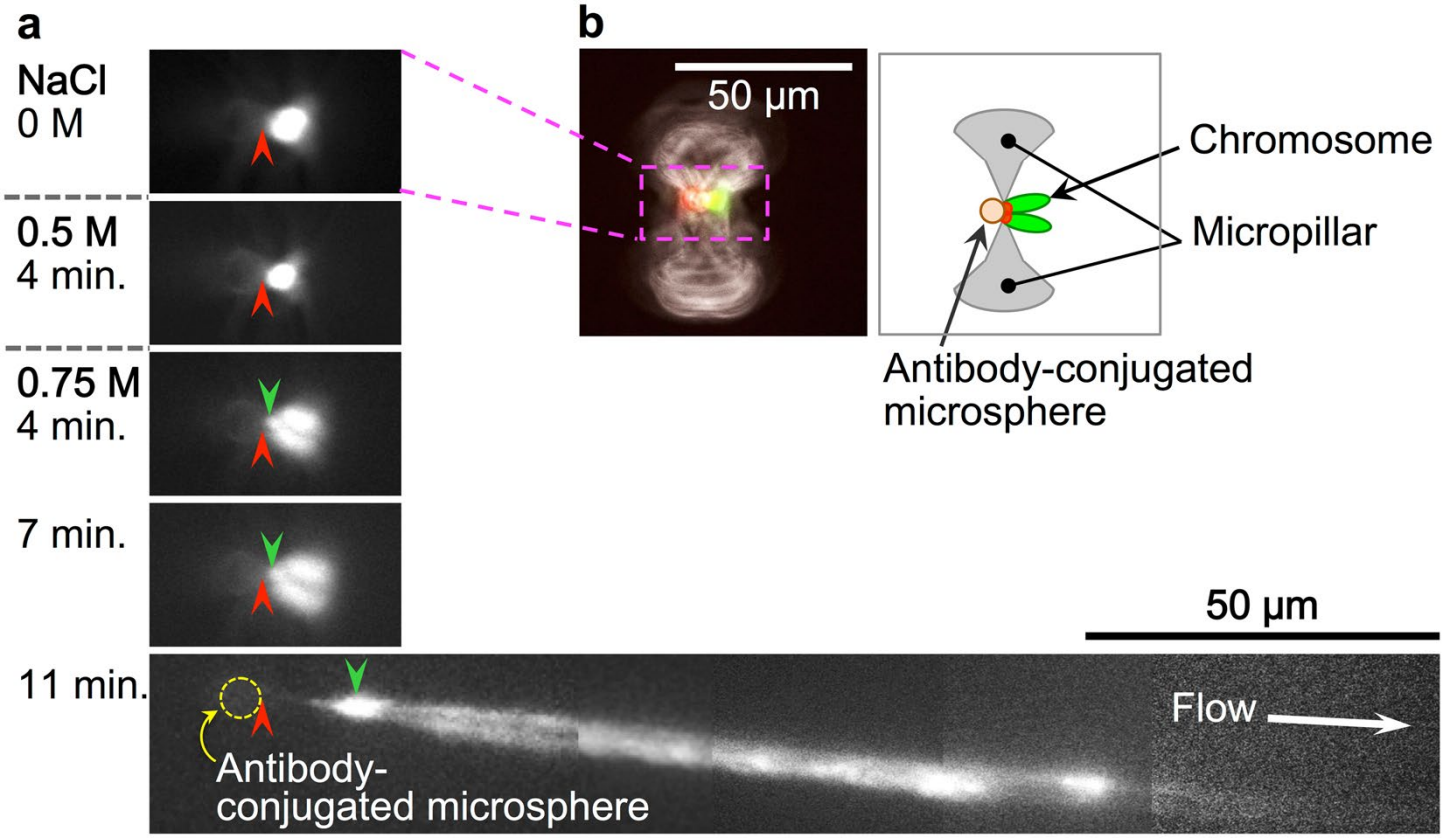
Change in the higher-order structure of a tethered mouse embryonic fibroblast (MEF) chromosome with stepwise increases in the salt concentration. (**a**) Fluorescence micrograph of a tethered chromosome visualised by staining with YO-PRO-1, a DNA dye. Red arrowheads indicate the site of attachment between the antibody-conjugated microsphere and chromosome, and green arrowheads indicate peri-centromeric regions. The time at which the new solution was changed was set to 0 min, with timekeeping reset at each solution change. The flow rate was ca. 100 µm/s. (**b**) Left: Pseudocoloured image of a chromosome tethered at the slit between micropillars via an antibody-conjugated microsphere in 0 M NaCl solution. DNA and methyl-CpG-binding domain-red fluorescent protein (MBD-RFP) were detected as green and red fluorescence, respectively, and are displayed in those colours; grey represents a phase-contrast image of the micropillars. Right: Schematic illustration of the tethered chromosome.

After tethering MEF chromosomes to the micropillars, we investigated their folding stability using stepwise increases in the salt concentration (Fig. 4). During the experiments, the flow rate in the main channels was maintained at around 100 µm/s by a water head difference between the solution in the inlet well and the waste solution connected to the outlet of the device via a silicone tube. In our microfluidic device, the channel distance between the solution inlet wells and the positions of tethered chromosomes was approximately 7–8 mm. Thus, it is estimated that tethered chromosomes were exposed to the new conditions within 2 min after solution exchange at the inlet well, considering the effect of the diffusion of solutes.

When the salt concentration of the solution introduced into the device was increased from 0 to 0.5 M, there was no significant change in chromosome morphology. Slight shrinkage of the chromosome was observed upon exposure to 0.5 M NaCl for 4 min (Fig. 4a), which was presumably an artefact of fluorescence visualisation resulting from a decrease in the binding constant of the cationic fluorescent DNA dye YO-PRO-1 at higher salt concentrations^34^. Nonetheless, when the concentration was further increased from 0.5 to 0.75 M, the chromosome gradually swelled, and the length of the long arms increased over time (Fig. 4a, from 4 to 7 min). The swollen chromosome began to unfold and stretch under the influence of the solution flow, and 11 min after the introduction of 0.75 M NaCl, both long arms were stretched to ca. 150 µm, almost 30 times their initial length (in 0 M NaCl). This drastic conformational change occurred at 0.75 M NaCl in all successfully tethered MEF chromosomes (N = 14). It should be noted that MEF chromosomes whose results are outlined above were prepared in a slightly different condition from that of ES cell chromosomes as described in Method. When isolation of MEF chromosomes was carried out under the same conditions as ES cell chromosomes (cells were incubated for 1 h in the presence of demecolcine without Latrunculin A treatment and subsequently washed with 0.5% Triton X after bursting, as described in the Methods section), we could not obtain dispersed MEF chromosomes (as they clumped together). In an alternative experiment, we tried to investigate salt-dependent morphological stability using non-dispersed MEF chromosomes, i.e., chromosomes clumped together in micropockets. However, we did not observe changes in morphology or apparent size even when these chromosomes were exposed to 0.5 M NaCl in the micropocket. On the other hand, when the salt concentration was increased to 0.75 M NaCl, the chromosomes gradually began to swell, and the morphology of individual chromosomes became unclear (Supplementary Figure S2). Thus, considering the range of conditions tested in this study, we established that neither treatment with demecolcine (for 1–4 h), the presence or absence of Latrunculin A, nor treatment with Triton X (0.5–1 wt%) had effect on the critical salt concentration at which the unfolding of the MEF chromosomes began.

Additionally, at 0.75 M NaCl, non-uniformity of fluorescence intensity along the stretched chromosome was observed, suggesting differences in folding stability along the length of the chromatin fibres. In a separate experiment, we observed further unfolding and stretching of the MEF chromosomes in the presence of 1.5 M NaCl; the chromosome length reached ca. 630 µm, almost 90 times the initial value (Supplementary Figure S3). Moreover, the stretched chromosome exhibited variations in the degree of folding along its length. Considering that the contour length of genomic DNA for the shortest chromosome in mouse (chromosome 19) is ca. 21 mm^35^, the observed length of 630 µm indicates that the chromatin is still highly compacted (more folded, on average, than the 10 nm fibre chromatin structure).

According to earlier studies, most MBDs dissociate from methylated DNA in a 0.5–0.8 M salt solution^36,37^. In this study, using 0.75 and 1.0 M NaCl solutions, most chromosomes detached from the anchored antibody-conjugated microspheres and flowed away, although some remained tethered, even in solutions containing up to 1.5 M NaCl. This was likely due to the formation of multiple bonds between MBD-RFP proteins in the peri-centromeric region and anti-RFP antibodies on the microsphere, which created steric hindrance that prevented chromosome dissociation.

### Folding stability of ES cell chromosomes

**Figure 5:**
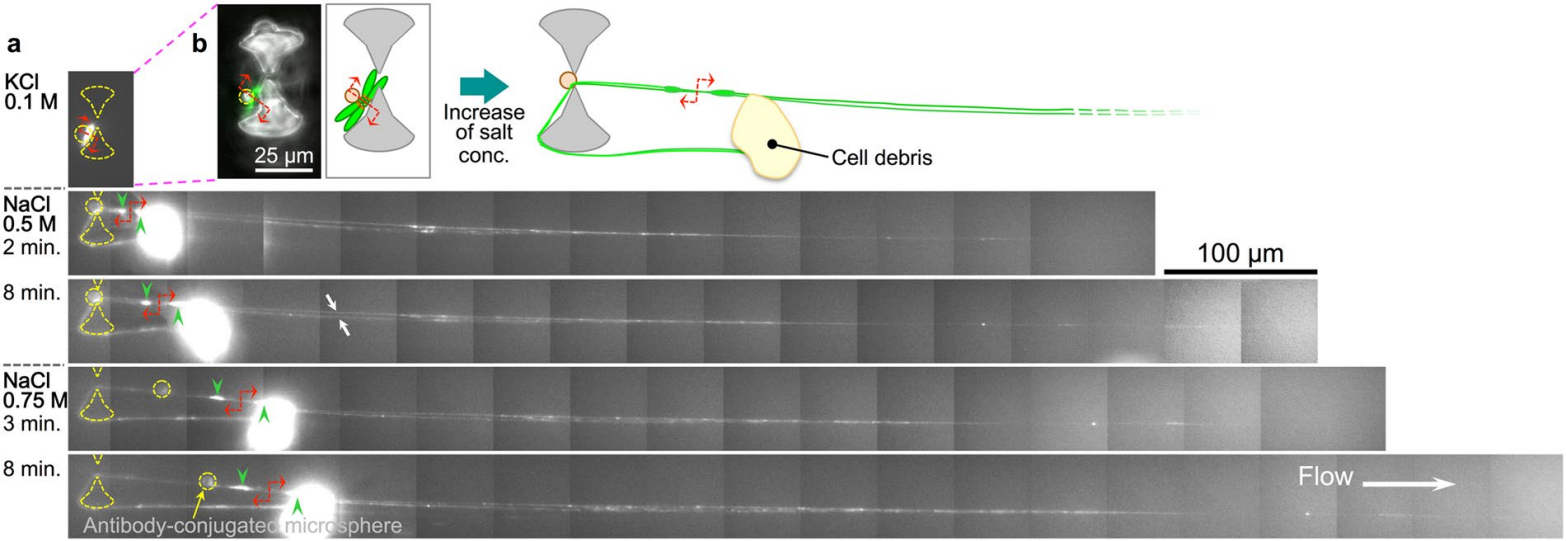
Change in the higher-order structure of tethered embryonic stem (ES) cell chromosomes with stepwise increases in the salt concentration. Two chromosomes were entangled via the short arm region by chance, and one peri-centromeric region was captured by the antibody-conjugated microsphere. (**a**) Fluorescence micrograph of tethered chromosomes visualised by YO-PRO-1 staining. The red double-headed arrow indicates the site of entanglement between the short arms of the two chromosomes. Stretching of one of the chromosomes was prevented by cell debris. Green arrowheads indicate peri-centromeric regions, and the white arrows (in 0.5 M, 8 min) indicate individual stretched long arms. The time at which the new solution was introduced was set to 0 min. The flow rate was ca. 100 µm/s. (**b**) Left: Pseudocoloured image of the chromosome tethered to the slit between micropillars by antibody-conjugated microspheres in the KCl solution. DNA was stained with YO-PRO-1 (green); micropillars and antibody-conjugated microspheres are shown in grey in the phase-contrast image. Right: Schematic illustration of tethered (0.1 M KCl, 0 min) and stretched (0.5 M NaCl, 8 min) chromosomes.

We examined changes in the higher-order structure of tethered ES cell chromosomes with stepwise increases in salt concentration (Fig. 5). Conformational changes, i.e., unfolding and stretching, occurred immediately upon exposure to the 0.5 M NaCl solution (This drastic conformational change occurred at 0.5 M NaCl in all successfully tethered ES cell chromosomes [N = 5]). This salt concentration was lower than that at which changes in MEF chromosome conformation were induced. In one representative case, contour length of the stretched chromosome was >700 µm (Fig. 5a, 0.5 M NaCl for 8 min), which was ca. 100 times the initial length. When the salt concentration was increased to 0.75 M NaCl, the chromosome stretched even further, to over 800 µm. Two strands of chromatin fibre from the long arms of daughter chromosomes were visible (white arrows), and bright fluorescent spots, i.e., highly folded regions, were distributed along the stretched chromatin fibres. However, since we were unable to distinguish chromosome number, we could not directly compare the differences in the distribution of fluorescent spots between stretched ES cell and MEF chromosomes of the same chromosome number. Nonetheless, ES cell chromosomes also exhibited variation in chromatin folding stability, although the sensitivity to the salt concentration differed between MEF and ES cell chromosomes.

## Discussion

The folding of ES cell chromosomes was sufficiently stable for manipulation under flow (≤100 µm/s) with the optically-driven microtool in the presence of 100 mM KCl. It has been reported that arrays of reconstructed nucleosomes show a compact form under quasi-physiological salt conditions (150–200 mM NaCl) relative to the apparent size of complexes seen under lower salt concentrations (≤50 mM NaCl)^38,39^. This folding of reconstituted chromatin comes from a reduction in the electrostatic repulsion between negatively charged chromatin fibres^38^. This explanation can be applied to our experimental results, i.e., the observed stabilization of ES cell chromosome folding. Thus, by direct micromanipulation and imaging of individual native chromosomes in a microchannel, we confirmed the folding stabilisation of chromosomes by tuning the salt concentration. Whereas chromosomes from both cell types exhibited the same highly-compacted morphology in M phase (Fig. 3a, 3b), ES cell chromosomes were relatively less stable under high-salt conditions (Fig. 5) compared to MEF chromosomes (Fig. 4), even though ES cell chromosomes were prepared with the Triton X solution containing 100 mM KCl for morphological stabilization. It has been reported that the chromatin-binding protein heterochromatin protein 1 (HP1) and histone H2B exhibit more hypermobility in mouse ES cells than in primary MEFs, as revealed by fluorescence recovery after photobleaching (FRAP) experiments^40^. Moreover, there was no difference in FRAP recovery of histone H1^0^-green fluorescent protein at any point in the cell cycle of mouse ES cells^41^. FRAP is powerful tool for investigating the turnover of chromatin-associated proteins in living cells^42,43^, although individual chromatin fibres cannot be distinguished owing to crowding within the nucleus. In addition, H1 and HP1 proteins in murine R1 ES, but not neural progenitor cells, were released at low salt concentrations^41^. These reports suggest that folding stability differs significantly between chromosomes from differentiated and undifferentiated cells and support our observations from single cell/single non-fragmented native chromosome-based experiments.

By comparing the degree of folding under 0.75 M NaCl conditions between MEF chromosomes (e.g., Fig. 4a, 0.75 M NaCl for 11 min) and ES cell chromosomes (e.g., Fig. 5a, 0.75 M NaCl for 8 min), it is likely that partially-unfolded MEF chromosomes maintained much higher-order folding structures than did ES cell chromosomes (for the comparison, an additional example is presented in the Supplementary information, Figure S4). This is consistent with previous findings which indicated that mouse ES chromatin shows greater fluctuations in spatio-temporal condensation within the nucleus than does MEF chromatin, which shows a more consistent distribution of chromatin condensation^44^. By single cell/single non-fragmented native chromosome-based experiments, we demonstrate that folding stability varies along the length of individual chromosomes, and that ES cell chromosomes exhibit greater stretching upon the replacement of a 100 mM KCl solution with one containing 0.5 M NaCl as compared to that of MEF chromosomes exposed to solutions with NaCl concentrations increasing from 0 to 0.5 M (Figs 4 and 5). Thus, ES cell chromosomes have more regions that may be easily unfolded, which may be related to the maintenance of a pluripotent state and their capacity to differentiate into any cell type.

In conclusion, we developed a method for investigating the distribution of folding stability along individual chromosomes at single-cell resolution without fragmentation. This system can serve as a powerful tool for evaluating the relationship between the progression of cell differentiation and changes in the distribution of folding stability along chromatin. Future studies will employ appropriate DNA sequence-specific fluorescence probes, such as dCas9-based probes^45,46^, to more clearly observe sites along tethered chromatin fibres and to discriminate between chromosomes, which will provide valuable information for epigenetic studies.

## Methods

### Cell culture and cell cycle arrest

Mouse embryonic fibroblasts (MEFs) and embryonic stem (ES) cells were obtained from a Methyl*RO* mouse expressing MBD-RFP^29^. Details pertaining to cell culture can be found in the Supplementary Material. MEFs were incubated for 4 h in the presence of 0.2 µg/ml demecolcine (Wako Pure Chemical Industries, Osaka, Japan), an inhibitor of tubulin filament polymerisation. Latrunculin A (Cayman Chemical, Ann Arbor, MI, USA; final concentration: 2 µM), an inhibitor of actin filament polymerisation, and Hoechst 33342 (Dojindo Laboratories, Kumamoto, Japan; final concentration: 5 µM) were added to the cells, followed by incubation for 30 min. ES cells were incubated for 1 h in the presence of 0.2 µg/ml demecolcine before adding Hoechst 33342 (final concentration: 5 µM) for 30 min. Following cell cycle arrest, cells were collected, and the solution was replaced with 300 mM sorbitol containing 5 μM Hoechst 33342 (referred to as isotonic solution in this manuscript). The cells were then immediately introduced into the microfluidic device for experiments. It should be noted that the cell cycle of MEFs is about 24 h, whereas that of ES cells is about 12 h. Therefore, in order to increase the yield of M phase cells, the incubation time of MEFs with demecolcine was made longer than that of ES cells (in fact, even in the case of treatment with MEF for 1 hour, cells in M phase was obtained, but acquisition efficiency was very low). We empirically determined the use of Latrunculin A and 1 wt% Triton X solution (composition is described in the next subsection) was optimal for obtaining dispersed MEF chromosomes that could be micro-manipulated individually using optical tweezers. In fact, under the determined conditions we managed to obtain a few dispersed chromosomes per cell (others remained clumped to each other).

### Antibody-conjugated microspheres, solutions for chromosome isolation, and investigation of structural stability

Antibody-conjugated microspheres were prepared as previously described^22^. Briefly, biotin-conjugated anti-RFP antibody (ab34771, rabbit polyclonal anti-RFP, Abcam, Cambridge, UK) and streptavidin-coated microspheres (diameter: 6 μm; PolyScience, Niles, IL, USA) were reacted to obtain anti-RFP antibody-conjugated microspheres that were dispersed in Triton X solution. The obtained microspheres were evaluated by fluorescence-labelled anti-rabbit IgG antibody (described in the Supplementary Material, Table S1). The hypotonic solution used to induce cell bursting was composed of 5 μM Hoechst 33342 and 30 mM dithiothreitol (DTT). The detergent solution for lysing the burst cell membrane (referred to as Triton X solution in this manuscript) was composed of 1 wt% (for MEFs) or 0.5 wt% (for ES cells) Triton X-100, 20 mM HEPES-KOH (pH 7.6), 1 mM EDTA, 30 mM DTT, and 0.5 µM YO-PRO-1. To investigate the stability of the higher-order structure of tethered chromosomes, Triton X solutions containing 0, 0.5, 0.75, 1, or 1.5 M NaCl were employed.

### Chromosome extraction in the microfluidic device

Selective use of the three ports of the microfluidic device reduced the influx of cell debris into the two main channels. First, the interior of the microfluidic device was filled with isotonic solution, after which the cell suspension was introduced into inlet well #1 or #2 (indicated in Fig. 1a), and sucked into the main channels. After placing cells in M phase into the micropockets by optical tweezers, the inlet well from which the cells were introduced was sealed using a silicone rubber sheet with a thickness of 1 mm. This was followed by introducing an isotonic solution from the other two inlet wells to flush out excess cells from the main channels. Hypotonic solution was then introduced from inlet well #3. By closing the inlet well used to introduce cells, influx of cell debris was reduced. Usually, when the inlet #1 was used for cell introduction, the main channel #2′ contained less cell debris, and when inlet #2 was used, the main channel #1′ contained less cell debris. Using two ports helped to prevent backflow in the main channels during solution exchange. The problem with backflow was that it usually dislodged the chromosome-attached antibody-conjugated microspheres from the tethering slits or caused entanglement of the tethered chromosomes/stretched chromatin fibres. The loading solutions, e.g., high-salt solutions, were replaced in each inlet well one by one. This procedure of solution exchange restricted backflow within the microfluidic channels to branch channels between the inlet ports and main channels.

### Experimental apparatus and conditions

The apparatus used for experiments, including an inverted fluorescence microscope (IX-73; Olympus, Tokyo, Japan) with a 100× oil immersion objective lens (UPlanFL N 100×, NA = 1.30, Ph 3), infrared laser for optical trapping (CW, λ = 1064 nm; IPG Laser GmbH, Burbach, Germany), and XY motorised stage (Sigma Koki Co., Ltd., Tokyo, Japan), has been previously described^23^. A high-sensitivity charge-coupled device (CCD) (C9100-13; Hamamatsu Photonics K.K., Hamamatsu, Japan) was used to capture images of low fluorescence intensity and an electron bombardment–CCD (C7190-43; Hamamatsu Photonics K.K.) camera was used to obtain relatively low magnification images with a higher fluorescence intensity. All experiments were performed at room temperature (298 ± 2 K).

## Electronic supplementary material

Supplementary Information

Movie 1

Movie 2

Movie 3

Movie 4
