# Supplementary material for: A microfluidic device for isolating intact chromosomes from single mammalian cells and probing their folding stability by controlling solution conditions
Source: Sci Rep. 2018 Sep 12;8:13684. doi: 10.1038/s41598-018-31975-5 (PMC6135817; doi:10.1038/s41598-018-31975-5)

Supplementary information

A microfluidic device for isolating intact chromosomes from single mammalian cells and probing their folding stability by controlling solution conditions

Tomohiro Takahashi[1], Kennedy O. Okeyo[2], Jun Ueda[3], Kazuo Yamagata[4], Masao Washizu[1,5], and Hidehiro Oana[1,*]

[1] Department of Mechanical Engineering, The University of Tokyo, Tokyo 113-8656, Japan

[2] Institute for Frontier Life & Medical Sciences, Kyoto University, Kyoto 606-8501, Japan

[3] Centre for Advanced Research and Education, Asahikawa Medical University, Asahikawa 078-8510, Japan

[4] Faculty of Biology-Oriented Science and Technology, KINDAI University, Kinokawa 649-6493, Japan

[5] Department of Bioengineering, The University of Tokyo, Tokyo 113-8656, Japan

*To whom correspondence should be addressed. Tel. & Fax: +81-3-5841-6338; Email: oana@mech.t.u-tokyo.ac.jp

1. Fabrication of the Microfluidic Device

The microfluidic device was fabricated by a standard soft lithography process (Ref. 24, in the main text). Briefly, a negative thick photoresist (SU-8 3025; MicroChem Corp., Westborough, MA, USA) was spin-coated to obtain a 30-μm-thick layer. The cycle of spin coating–exposure–development was performed twice to obtain a double-layered mould with a total thickness of 60 μm. Then, the PDMS chip (Figure 1b, in the main text) was fabricated using the mould and bonded to a coverslip to form microchannels. After the fabrication of the inlet and outlet, 1 wt% bovine serum albumin (BSA) (dissolved in ultrapure water) was introduced into the microchannels, and at least 3 h was allowed for surface modification inside the device to prevent non-specific attachment of cells, isolated chromosomes, and antibody-conjugated microspheres. Then, the BSA solution in the microchannels was flushed with a 300 mM sorbitol solution to achieve an isotonic condition inside the device prior to the introduction of cells.

2. Size and Layout of the Microstructures in the Microfluidic Device

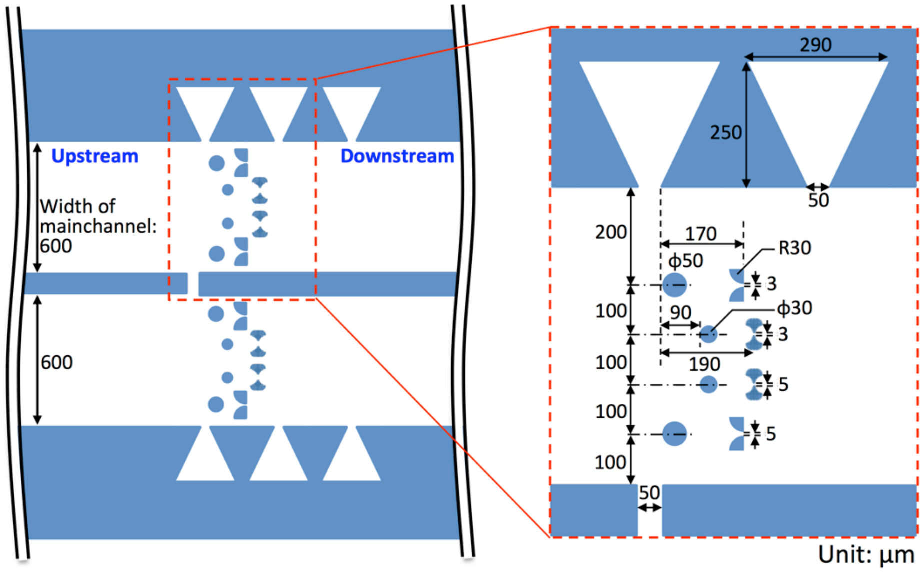

Figure S1. Size and layout of the microstructures in the two main channels. The gap between the micropillars was set to 3 or 5 μm, which was smaller than the diameter of the antibody-conjugated microspheres.

3. Culture of MEFs

The mouse embryonic fibroblasts (MEFs) were cultured in Dulbecco's Modified Eagle's Medium (DMEM; Thermo Fisher Scientific, Waltham, MA, USA; 11995-065), 10% foetal bovine serum (FBS; Thermo Fisher Scientific; 16000-044), and 1% penicillin-streptomycin. Cells were passaged every 2–3 days and synchronised in M phase when they reached 50%–70% confluence.

4. Culture of ES cells

The medium for embryonic stem (ES) cell culture included StemSure DMEM (Wako Pure Chemical Industries, Osaka, Japan; 197-16275), 15% FBS (Thermo Fisher Scientific; 16000-044), 1× Minimal Essential Medium with Non-essential Amino Acid Solution (Life Technologies, Carlsbad, CA, USA; 11140-050), 1× GlutaMAX Supplement (Life Technologies; 35050-061), 0.1 mM 2-mercaptoethanol (Nacalai Tesque, Kyoto, Japan; 214-18), 1000 U/ml leukaemia inhibitory factor (Wako Pure Chemical Industries; 129-05601), 20 µg/ml gentamicin (Sigma-Aldrich, St. Louis, MO, USA; G1397-100ML), 3 µM CHIR99021 (Sigma-Aldrich; SML1046), and 1 µM PD0325901 (Sigma-Aldrich; PZ0162). The first seven components were mixed, and long-term storage of the solution was performed at 4 °C; CHIR99021 and PD0325901 were added prior to use. The medium was used within 2 weeks of adding these last two components. ES cells were passaged every 2 days and synchronised in M phase when they reached 50%–70% confluence.

5. Evaluation of anti-RFP antibody-conjugated microspheres

Conjugated antibody to the microsphere[1)]	Binding ability to chromosomes from Methyl*RO* mouse	Immunofluorescence signal using anti-rabbit IgG antibody[4)]
**Anti-RFP[2)]**	**Yes**	**Yes**
Anti-GFP[3)]	No	Yes
None	No	No

1) Streptavidin microspheres, 6.0µm (Polysciences, Inc.).

2) Anti-RFP antibody (Biotin) (ab34771, Abcam), rabbit polyclonal antibody.

3) Anti-GFP, rabbit IgG fraction, biotin-XX conjugate (A10259, Invitrogen), rabbit polyclonal antibody.

4) Goat Anti-Rabbit IgG H&L (Alexa Fluor® 405) (ab175652, Abcam).

Table S1. Evaluation of anti-RFP antibody-conjugated microspheres. The ability of anti-RFP antibody-conjugated microspheres, anti-GFP antibody-conjugated microspheres, and streptavidin-coated microspheres to bind to chromosomes obtained from Methyl*RO* mouse cells was investigated and confirmed by using optical manipulation (contact and pull) of the microspheres. Of the three, only anti-RFP antibody-conjugated microspheres were able to attach to the chromosome involved with methyl-CpG-binding domain-red fluorescent protein (MBD-RFP). In addition, immunofluorescence staining against rabbit IgG showed that anti-RFP antibody-conjugated microspheres and anti-GFP antibody-conjugated microspheres were conjugated with rabbit IgG proteins. These results show that anti-RFP antibody-conjugated microspheres were prepared successfully with the specific ability to bind to chromosomes through MBD-RFP.

6. Folding/morphological stability of MEF chromosomes isolated from MEF incubated for 1 h in the presence of demecolcine without Latrunculin A treatment and washed with 0.5% Triton X after the cell bursting

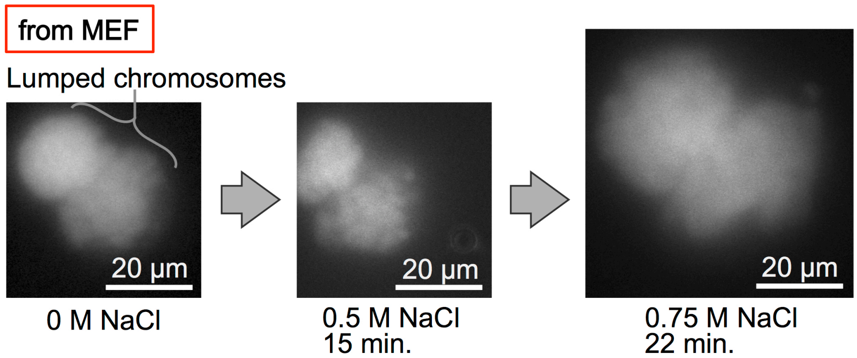

Figure S2. Representative fluorescence (YO-PRO-1) micrograph of extracted mouse embryonic fibroblast (MEF) chromosomes in the micropocket. Left: MEF chromosomes in 0.5 % Triton X. Centre: MEF chromosomes exposed to 0.5 M NaCl for 15 min. Significant changes in morphology and size are not observed. Right: MEF chromosomes exposed to 0.75 M NaCl for 22 min. Chromosomes are swollen and the individual morphology of the chromosomes became unclear.

7. Stretching of the MEF chromosome in the presence of 1.5 M NaCl

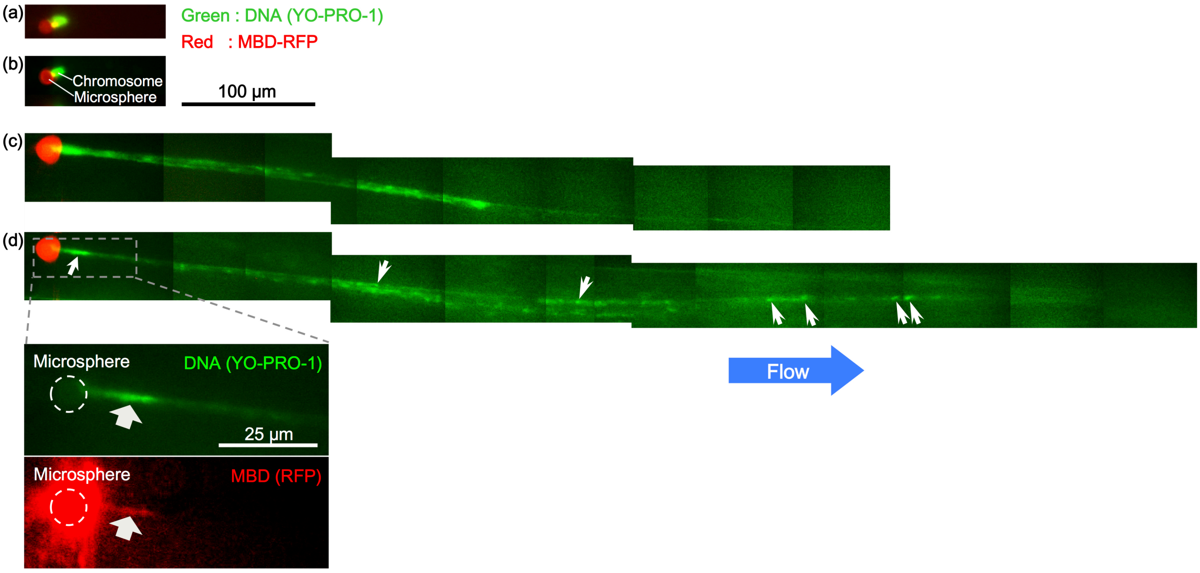

Figure S3. Representative fluorescence micrograph of an unfolded and stretched MEF chromosome in the presence of (a) 0 M NaCl, (b) 5 min after the introduction of 0.5 M NaCl, (c) 25 min after the introduction of 1 M NaCl, and (d) 20 min after the introduction of 1.5 M NaCl. DNA was stained with YO-PRO-1 (green fluorescence) and MBD-RFP was visualised by red fluorescence. Multiple folded/unfolded regions were observed along the extended fibre (representative folded regions are indicated by white arrows in panel d). Bottom: Magnified image of the area around the tethering point in panel d. Green fluorescence intensity was high at the peri-centromeric region captured by the microsphere, indicating that the chromatin fibre was still highly compact. Red fluorescence was also visible at the peri-centromeric region, indicating the presence of hypermethylated DNA.

8. Comparison of salt-dependent stretching in MEF and ES cell chromosomes

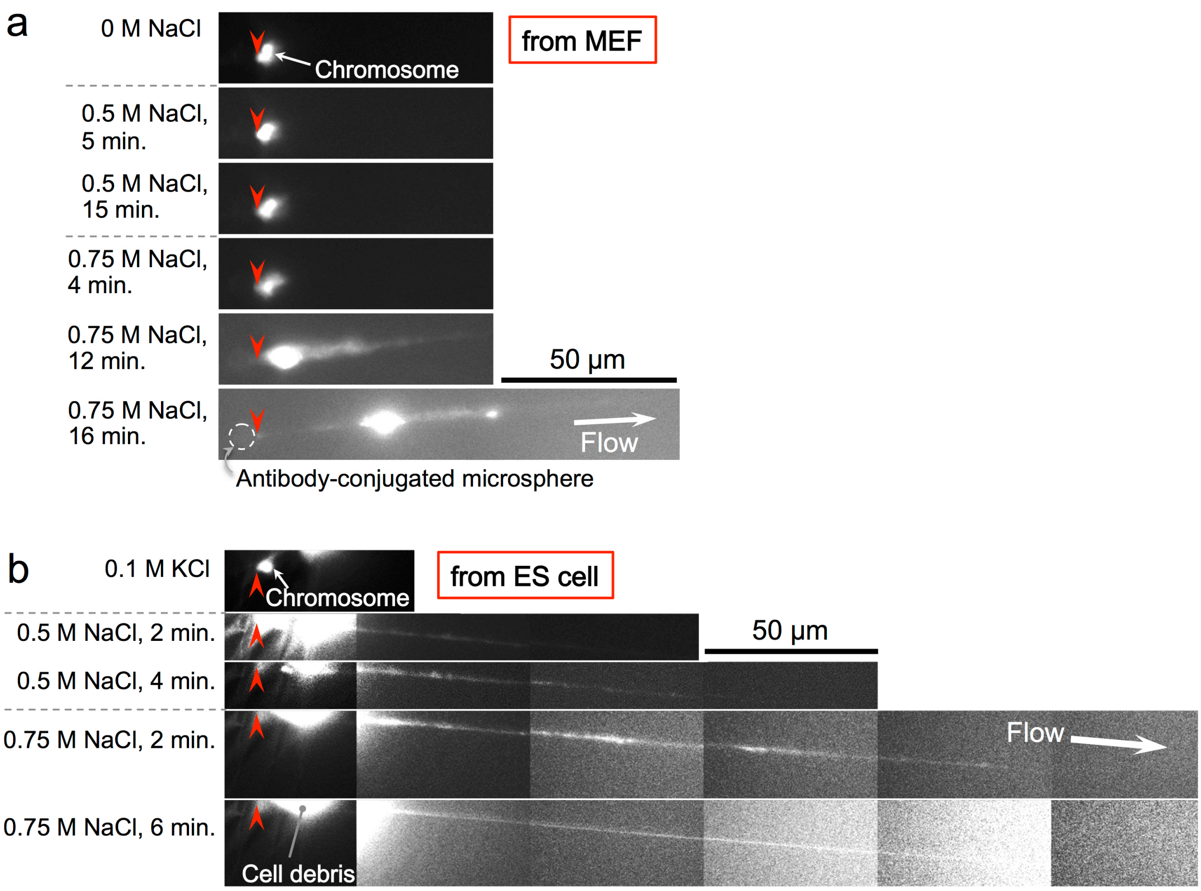

Figure S4. Representative fluorescence image of chromosome/chromatin fibres visualised by YO-PRO-1. (a) Unfolding of mouse embryonic fibroblast (MEF) chromosome with increasing NaCl concentration. Chromosome unfolding and stretching started at 0.75 M NaCl. (b) Unfolding of chromosome obtained from an embryonic stem (ES) cell with increasing NaCl concentration. Chromosome unfolding and stretching started at 0.5 M NaCl. It is likely chromosomal folding was of a much higher order in partially unfolded MEF chromosomes maintained than in ES cell chromosomes.

9. List of movies

Movie 1. Chromosome extraction from a MEF in a micropocket (at 2× speed). The MEF in a micropocket was visualised by phase contrast microscopy. When a hypotonic solution was introduced into the main channel, the cell started to swell due to the reduction in osmotic pressure and finally burst, and cellular contents flowed out (this movie starts ca. 2 min after introduction of the hypotonic solution). Isolated chromosomes were identified by switching to fluorescence microscopy (last ca. 8 s, of the movie, detection of Hoechst33342).

Movie 2. MEF chromosome translocation and tethering (real-time movie). An antibody-conjugated microsphere and a chromosome made contact and stable binding was established within several seconds. After the capture, the fluorescence filter was switched to visualise YO-PRO-1 fluorescence (8 s), and translocation was initiated (bight field illumination was also used to visualise the PDMS microstructures). The captured chromosome was dragged out of the micropocket and moved to the micropillar region in the main channel, then moved to a slit between the micropillars upstream of the main channel; the flow in the main channel (from left to right in the frame) induced the passage of the chromosome through the slit. Optical trapping was terminated and the microsphere was moved and immobilised between the pair of micropillars under flow to achieve chromosome tethering. At the end of the movie, bright field illumination was turned off and the tethered chromosome can be clearly seen based on the fluorescence signal of YO-PRO-1.

Movie 3. Unfolding of ES cell chromosomes during translocation in the absence of salt (real-time movie). After isolation, ES cell chromosomes exposed to Triton X solution (without salt) showed slight swelling (Right: Typical fluorescence image of extracted ES cell chromosomes exposed to Triton X solution [without salt] in the micropocket.). Then, when the chromosome was dragged using optical tweezers, the chromosome became unfolded and stretched.

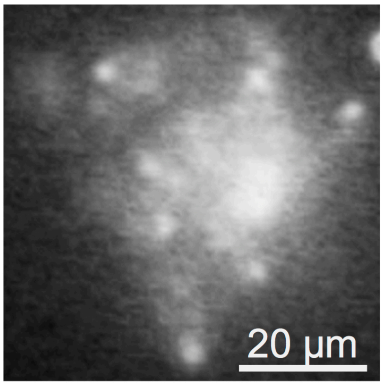

Movie 4. Stable morphology of ES cell chromosome during dragging in the presence of salt (real-time movie). Isolated ES cell chromosomes exposed to Triton X solution containing 100 mM KCl. did not show swelling (Right: Typical fluorescence image of extracted ES cell chromosomes exposed to Triton X solution containing 100 mM KCl in the micropocket). After that, the chromosomes did not unfold when dragged.

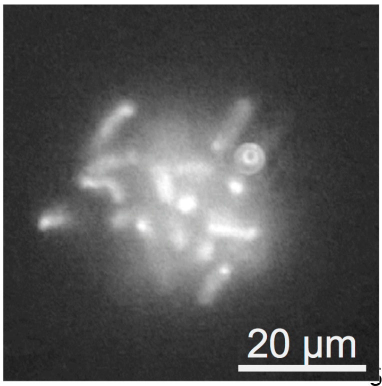

Supplement: Supplementary file 1 — Supplementary Information [file 41598_2018_31975_MOESM1_ESM.pdf]
